# Significant Interfacial Dielectric Relaxation of Covalently Bonded Ice-Hydrogels

**DOI:** 10.3390/gels8070409

**Published:** 2022-06-28

**Authors:** Yongqiang Li, Liufang Chen, Chuanfu Li, Lin Lin, Zhibo Yan, Junming Liu

**Affiliations:** 1 Laboratory of Solid State Microstructures, Nanjing University, Nanjing 210093, China; lfchen502@163.com (L.C.); lichuanfu_com@126.com (C.L.); liujm@nju.edu.cn (J.L.); 2 Department of Applied Physics, College of Science, Nanjing Forestry University, Nanjing 210037, China; llin@njfu.edu.cn

**Keywords:** chitosan, ice-hydrogels, dielectric relaxation behavior, activation energy, interface

## Abstract

Hydrogels are composed of a three-dimensional network of cross-linked hydrophilic polymer chains and large amounts of water. The physicochemical properties of the polymer-water interface in hydrogels draw our attention. Due to the complex structure of hydrogel systems, it is still a challenge to investigate the interfacial layer properties of hydrogels through experiments. In this work, we investigate the properties of the covalently bonded chitosan-based ice-hydrogels interfacial layer by dielectric relaxation spectroscopy (DRS) techniques in the presence of avoided electrode polarization. The DRS data exhibit that the polymer-water interfacial layer has a strong dielectric signal response, which indicates that a large number of polar electric dipoles or polar molecules may be contained in the interfacial layer. The variable temperature dielectric relaxation behavior of a series of chitosan-base ice-hydrogels showed that the value of dielectric activation energy for different water contents is about 180 kJ/mol, which is much larger than that of the polymer and ice phases, suggesting a strong coupling of polar electric dipoles within the interfacial layer. This work demonstrates the important role of the polymer-water interface in covalently bonded hydrogels, which will provide assistance in the design and application of covalently bonded hydrogels.

## 1. Introduction

Hydrogels are composite materials consisting of hydrophilic three-dimensional polymer networks and lots of water [1]. These polymer networks are crosslinked through covalent chemical bonds and/or physical interactions. Due to the inherent softness, deformability, biocompatibility, and reversible stimulus response to external stimuli, hydrogels are considered to be the most compatible materials with natural extracellular matrices in terms of physical, chemical, and biological properties [2,3]. There is no doubt that hydrogels have been widely used in areas including drug delivery, cell culture, and tissue engineering [4,5].

Among the hydrogel family, natural polymer hydrogels have received a lot of attention due to the rich source of natural polymers, low cost, excellent biocompatibility, and biodegradability, etc. [6]. As the only natural cationic polymer [7], chitosan (CS) not only has the physicochemical properties possessed by natural polymers, but also has abundant amine groups (-NH_2_) and hydroxyl groups (–OH) along the chain, which can easily interact with related substances to form chitosan (CS)-based hydrogels [8,9]. The CS-based hydrogels allow responses to various environmental stimuli, such as pH responses [10], temperature responses [11], ionic responses [12], and electric field responses [13]. Therefore, CS-based smart hydrogels are not only limited to a wide range of applications in the biomedical field [8] but have been deeply explored in the area of flexible electronics [14,15]. Undoubtedly, a comprehensive knowledge of these functions of hydrogels is of great significance, both in terms of the design and application.

**Figure 1:**
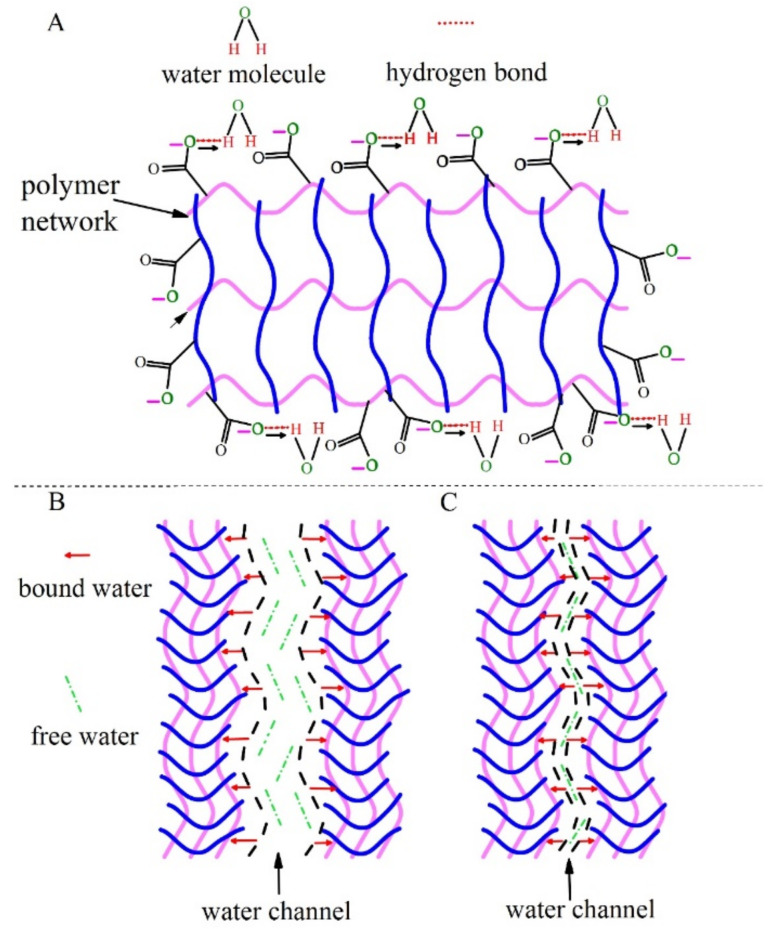
(**A**) Schematic diagram of the structure of chitosan-based hydrogel network. (**B**) High water content hydrogels network with water channels filled with free water. (**C**) In the low water content hydrogels network, the narrow water channels are filled with very little free water.

It is worth mentioning that the physical and chemical properties of CS-based hydrogels, as well as the origin of the microstructure, have been extensively studied for decades. One approach addresses the issue from the composition of the CS-based hydrogels combined with the microstructure. The mainstream in this discipline seems to be related to the water phase and polymer network as two separate or independent components of the microstructure, although the hydrophilic or hydrophobic nature of the polymer phase can be an issue under consideration. Thus, the physical and chemical properties are more or less relevant with the optimization and modulation of the polymer network from a chemical and functional viewpoint, while the water phase plays a role basically as a filler. What we are addressing here is the polymer-water interfacial coupling that has been relatively less understood but could be non-negligible or even very important in many cases. In particular, for covalent bond-dominant hydrogels, the interfacial coupling should be strong enough to influence the properties of the hydrogel system. Among the covalent CS-based hydrogels, semi-interpenetrating network hydrogels have become a hot topic of research with issues such as porosity, elasticity, degree of swelling, and response to stimuli. Here, we take chitosan/polyacrylic acid (CS/PAA) hydrogels as an example since they are one of the most studied semi-interpenetrating network hydrogels. A schematic diagram in Figure 1A shows the structure of semi-interpenetrating network CS-based hydrogels, featuring the polymeric network as micro pores and water filling these pores. Along this line, at least several issues should be highlighted.

First, the spatial structure of hydrogels depends remarkably on the water content, and thus the as-generated functionalities may vary considerably, too. This also applies to the CS-based hydrogels. It is believed that the fill water can be divided into free water and bound water [16]. The free water phase exists in the micro pores of hydrogels, while the bound water molecules are chemically bound with chemical groups of the polymer, e.g., –COOH, –NH_2_, and –OH etc., as shown in Figure 1. Here, for convenience in Figure 1A, only –COOH groups bounded with water molecules through the hydrogen bonding are drawn for illustrating the bound water phase. Therefore, a comprehensive understanding of the bound water phase becomes urgently required, in particular the interfacial properties between the binding groups and bound water molecules.

Second, one looks at the possible mechanical behavior of hydrogels. For one thing, water plays an important role in hydrogels by supporting their integrity, solubility, and diffusion of substances. For another thing, the water-wetness and softness of the CS-based polymer allow its sensitive response to external force. Consequently, the polymer network deformation is accompanied by the water droplet shape and size. This deformation as a core property for hydrogel applications depends on not only the water content and polymer network structure, but also the polymer-water interfacial coupling, while this coupling would be a major source for the formation of bound water phase. For instance, when the water content is high, the polymeric network will be swollen, and then the water channels will be wide and filled mainly with free water phase, as shown in Figure 1B. On the contrary, what is demonstrated in Figure 1C is that at low water content, the polymeric network will be partially collapsed, leading to the water channel shrinking. It is believed that the water channels would be filled mainly with the bound water phase, making the mechanical behaviors greatly different.

Ending with the two-aspect discussion, it is clear that the polymer-water interface should be an emergent object for subsequent investigation. In fact, the study of the polymer-water interface of hydrogels is involved from the basic mechanical properties of hydrogels to the applications. On the one hand, as we know, polyampholyte (PA) hydrogels are designed with strong bonds as permanent cross-linking points and weak bonds as reversible sacrificial bonds, resulting in excellent mechanical strength and toughness of these hydrogels [17]. Xing et al. introduced a thermodynamic approach to understand the unique mechano-chemo-electrotaxis coupling and interfacial dynamics in PA hydrogels to explain the excellent mechanical properties of the hydrogels [18]. It was concluded that the interfacial bonding strength is the key factor affecting the mechanical strength and reversibility of reconstruction of the PA hydrogel system. On the other hand, in order to develop potential applications of hydrogels including stretchable conductors [19], ionic cables [20], and neuro-prostheses [21], in-depth understanding and systematic research of the thermal transport mechanism in hydrogels are required for researchers. By building a theoretical model, Xu et al. calculated when the water fraction of hydrogels is under 85%, the thermal conductivity can be even higher than the thermal conductivities of both pure polymer networks and pure water due to the influence of the interface between polymer networks and water [22]. The theoretical results agree well with the results simulated by treating the hydrogel as a three-phase (polymer phase, water phase, and interface phase) composite model.

The existence of the polymer-water interface in hydrogels was demonstrated through theoretical modeling and plays an important role in the mechanical properties and functional applications. Although there are many challenges to study the interfacial properties of hydrogels by experimental means, we have successfully investigated the interface of predominantly non-covalent polyacrylonitrile (PAN)-based ice-hydrogels using dielectric relaxation spectroscopy (DRS) techniques [23]. In the interfacial studies of hydrogels, electron polarization (EP) effect is the challenge that must be faced because the EP effect produces a huge dielectric constant that can overwhelm the inherent signal of the hydrogels. The EP effect was avoided by measuring ice-hydrogels instead of water-hydrogels in the research. The main results of PAN-based hydrogels are shown in two aspects. On the one hand, the ice-hydrogels exhibit a significant contribution in dielectric relaxation from the interfacial layer. On the other hand, the dielectric activation energy is estimated to be 50–60 kJ/mol, which is similar to that of ice, indicating that the electric dipole coupling of the interfacial layer is weak.

We study the properties of the interface of covalent hydrogels along the lines of non-covalent hydrogels. For covalently bonded CS-based hydrogels, the chemical composition and structure are completely different from non-covalent PAN-based hydrogels. In this work, we shall measure the dielectric response of the polymer-ice interfacial layer of covalent CS-based ice-hydrogels, focusing on the electro-relevant dynamics for the polymer-water interfacial coupling, as characterized by the activation energy (*E_a_*) for the polarizable molecules in the interfacial layer, which can be evaluated upon different water contents. Meanwhile, we compared the covalent hydrogels and non-covalent hydrogels data and found significant differences, which is mainly reflected in two aspects. For one thing, the dielectric relaxation strength (dielectric value) of the interfacial layer of the covalent CS-based ice-hydrogels should be smaller than that of the non-covalent PAN-based ice-hydrogels, implying the electric dipole density in the interfacial layer of the covalent bond is lower. For another, the activation energy of the covalent bond should be larger than that of the non-covalent bond, which indicates that the electric dipole coupling of the interfacial layer is stronger. The present work thus represents a substantial step forward towards deep understanding of the electrostatic behavior of the polymer-water interfacial layer, a subject less touched so far.

## 2. Results and Discussions

### 2.1. Structures and Bonding

**Figure 2:**
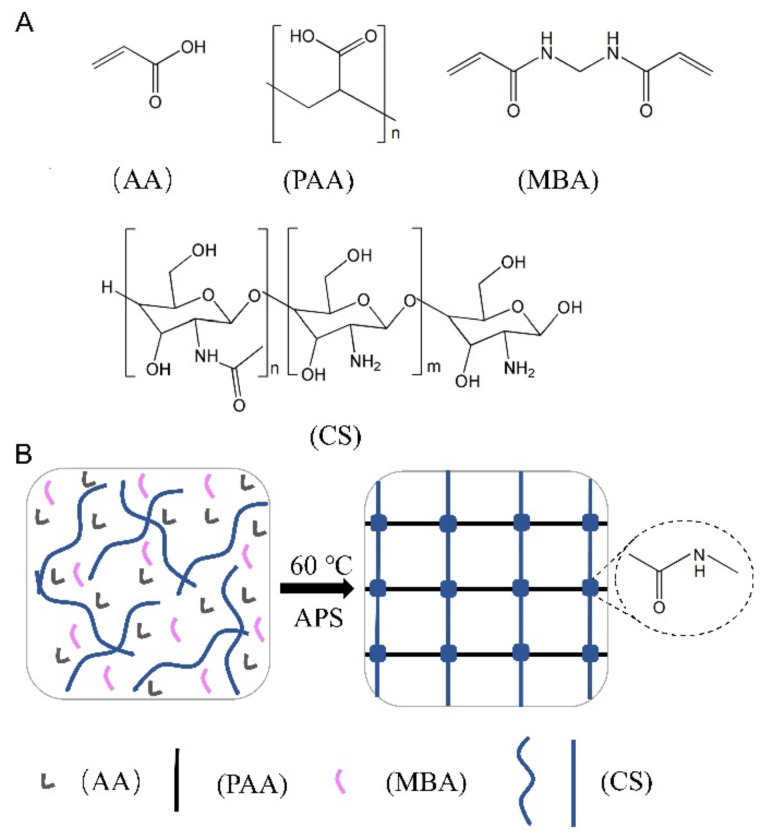
(**A**) Structural formulas of acrylic acid (AA), polyacrylic acid (PAA), chitosan (CS), and N, N′-Methylenebis(acrylamide) (MBA). (**B**) Schematic diagram of the one-pot method for the synthesis of CS-based hydrogels.

The materials and reaction principles used in the hydrogel synthesis are first introduced, as shown in Figure 2. In our experiments, the raw materials were used in the synthesis of CS-based hydrogels which include the chitosan (CS), acrylic acid (AA), Ammonium persulfate (APS), and N, N’-Methylenebis (acrylamide) (MBA). The AA, polyacrylic acid (PAA), MBA, and CS of chemical configurations are schematically drawn in Figure 2A.

The CS-based hydrogels were synthesized via a one-pot method and a simple sketch of the synthesis is shown in Figure 2B. As can be seen from the schematic diagram, in the presence of the cross-linking agent MBA and the initiator APS, and under heating conditions, the covalently cross-linked CS-based hydrogels were synthesized by the reaction of monomer CS and AA. During the hydrogel synthesis, the reaction of the –NH_2_ group in CS with the –COOH group in AA will form the -CO–NH- group, which indicates the successful synthesis of chitosan-based (CS/PAA) hydrogels.

**Figure 3:**
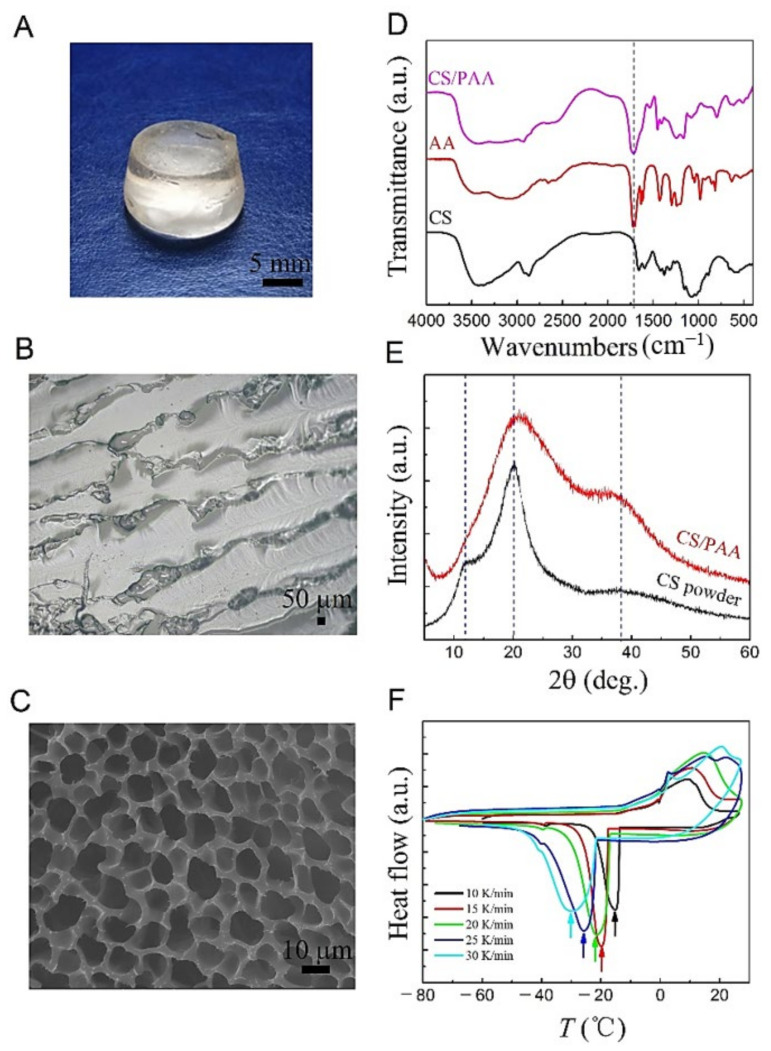
(**A**) Photos of the wet-hydrogels (*f_w_* ~ 0.73). (**B**) Optical photograph of the wet-hydrogels microstructure. (**C**) ESEM image of the dried hydrogels morphology. (**D**) FTIR spectra of chitosan (CS) powder, acrylic (AA) liquid, and dry chitosan-based hydrogels samples. (**E**) XRD patterns of chitosan (CS) powder and dried chitosan-based hydrogels samples. (**F**) DSC curves of chitosan-based hydrogels samples measured in cooling-heating cycles with cooling/heating rates of 10 K/min, 15 K/min, 20 K/min, 25 K/min, and 30 K/min, respectively.

From a macroscopic point of view, the as-prepared cylindrical hydrogel sample is shown in Figure 3A, where the shape of the hydrogel is slightly changed due to the dissolution equilibrium in water. The as-prepared hydrogels are colorless and transparent with the water content of *f_w_* ~ 0.73. To understand the microstructure of hydrogels, wet-hydrogels and dry-hydrogels were investigated separately. The microstructure of the wet-hydrogels observed by optical microscopy is shown in Figure 3B, which demonstrates several channels surrounded by the polymer that should be filled with a specific amount of water. The size of the channels is relatively uniform with the average size of 100 μm. The microstructure of the wet-hydrogels is good and consistent with the schematic diagram of the hydrogels depicted in Figure 1. The ESEM was employed to observe the microstructure of dry-hydrogels exhibited in Figure 3C, which displayed many micro pores and uniform size with the average dimension of about 5 μm. According to Figure 3B,C, we can get a more three-dimensional understanding of the microstructure of the CS-based hydrogels, which will help us to carry out the next work.

For the chemical bonding of CS-based hydrogels, the FTIR data is exhibited in Figure 3D, where CS and AA of data is inserted for comparison. The FTIR data for CS mainly includes two vibrational modes, where the C=O group at 1660 cm^−1^, the C–N group at 1379 cm^−1^, and the C–O–C group at 1074 cm^−1^ are tensile vibrational modes, while the N–H group at 1579 cm^−1^ is a bending vibrational mode [24]. The characteristic peak of AA is the –COOH group coming from 1720 cm^−1^, which is the stretching vibrational mode. However, from the FTIR data of CS/PAA hydrogels, it is observed that the pattern of N–H groups at 1597 cm^−1^ disappears and the patterns of C=O and C–O–C groups at 1660 cm^−1^ and 1074 cm^−1^ are severely suppressed [25]. Instead, additional stretching vibrational modes from the COO– group at 1540 cm^−1^ and 1410 cm^−1^ were detected, in addition to the –COOH group at 1720 cm^−1^, which was derived from AA. These features suggest that AA has been successfully grafted onto CS chains to synthesize our designed CS/PAA hydrogels.

The crystallization of the as-synthesized CS/PAA hydrogels was examined by XRD, the result of which is shown in Figure 3E. The XRD pattern of CS powder was first discussed, from which two distinct diffraction peaks at 11.7° and 19.9° were observed, indicating that CS powder has good crystallinity [26]. For the CS/PAA hydrogel, it can be observed from the XRD spectrum that the diffraction peak of CS powder at 11.7° is difficult to observe due to the weak crystallinity, while the position of the diffraction peak at 19.9° moves to 20.8°, accompanied by the increase of diffraction peak area and the decrease of diffraction intensity, and an amorphous diffraction peak appears at 38.3°. The successful synthesis of CS/PAA hydrogels was also indirectly demonstrated from the XRD spectra. These characteristics indicate that the strong hydrogen bonds between the CS molecules and the regular arrangement of the chains are disrupted, leading to the reduction in the crystallization ability due to cross-linking between the polymer chains limiting the mobility [27]. Although the crystallinity of the synthesized hydrogels is reduced, the CS chains are still dominant in the hydrogels, which is consistent with the interfacial coupling theme of CS-based hydrogels.

Although the polymer phase is dominated by the amorphous state, the DSC data can demonstrate the existence of crystalline ice phase in as-prepared ice-hydrogels, even though the water phase is separated into small-sized droplets by the polymer network. The DSC data with five different cooling/heating rates (10, 15, 20, 25, and 30 K/min) are shown in Figure 3F. Although the ice-hydrogels melting temperatures at different rates display broad peaks at approximately the same location, the exothermic peak during solidification of the hydrogels presents differently. It is mainly manifested in two aspects: firstly, the slower the cooling rate, the sharper the exothermic peak, and the wider the width of the peak shape as the cooling rate increases; secondly, as the cooling rate increases, the peak moves to the low temperature side, which is typical of the liquid water crystallization and solidification process. This indicates that the ice phase crystallizes well despite the amorphous nature of the hydrogels due to polymer network limitations.

### 2.2. Significant Interfacial Layer Contribution

Having understood the microstructure of CS-based hydrogels, we now provide clear experimental evidence that the interfacial contribution can be studied by DRS, so that the interfacial phase between the polymer phase and the water phase should also be considered when studying the properties of hydrogels. The microstructures of hydrogels are shown in Figure 3B,C, and the hydrogels can be regarded as composite materials consisting of the polymer phase and the water phase. The Maxwell–Garnett equations can represent the dielectric constant *ε_r_* of the two-phase structure [28]:

(1) $$\varepsilon_{r}=\varepsilon_{p}\left(1+\frac{df_{w}\beta }{1-f_{w}\beta }\right),\;\beta =\frac{\varepsilon_{w}-\varepsilon_{p}}{\varepsilon_{w}+(d-1)\varepsilon_{p}},$$

where the *ε_p_* is the real dielectric part of the polymer, *ε_w_* is the real dielectric part of the ice phase, *f_w_* represents the volume fraction occupied by the ice in the ice-hydrogel, and *d* (*d* = 3) refers to the space dimensionalities of the material structure.

**Figure 4:**
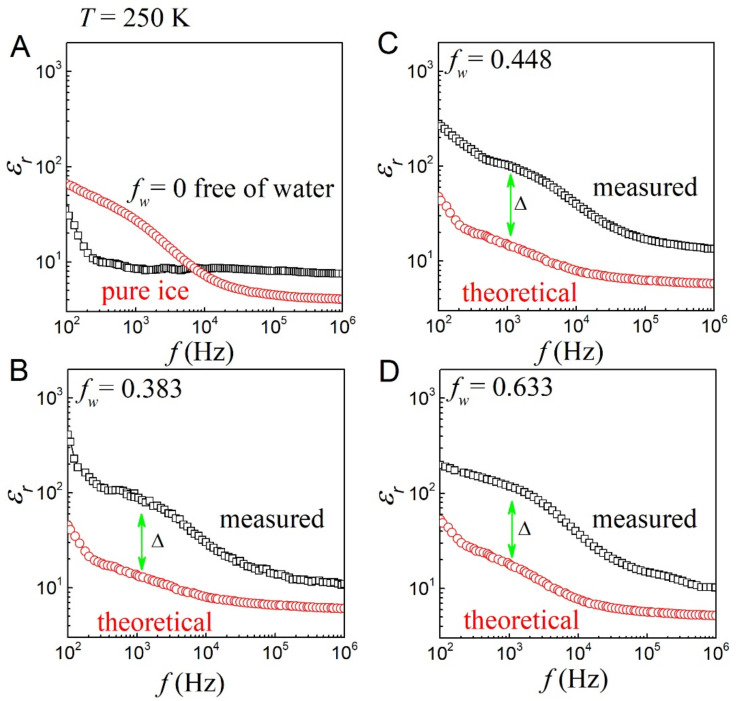
Dielectric constant *ε_r_*(*f*) vs. frequency (*f*) curves for all hydrogels at *T* = 250 K. (**A**) *ε_r_*(*f*) curves of pure ice and dry chitosan-hydrogels. (**B**–**D**) Measured permittivity *ε_r_*(*f*) (black open squares) and theoretical permittivity *ε_r_*(*f*) (red open circles) for the hydrogels with *f_w_* of 0.383, 0.448, and 0.633, where the symbols Δ represent the difference between the measured and theoretical permittivity *ε_r_*(*f*) in the low and mid frequency range.

The dielectric constants (*ε_r_*) of pure ice and dried CS-based hydrogels were measured, where a set of data with *T* = 250 K and the frequency range of 100 Hz to 1.0 MHz is plotted in Figure 4A. According to the frequency, their dielectric data can be divided into three regions for discussion. Firstly, the values of pure ice and dry hydrogel are *ε_w_* ~ 60 and *ε_p_* ~ 30 in the low frequency range, respectively; secondly, pure ice exhibits a strong frequency dispersion in the medium frequency range, while dry hydrogels indicate a weak frequency dependence; finally, pure ice and dry hydrogels show very weak frequency dependence in the high frequency range with the values of *ε_w_* ~ 4.0 and *ε_p_* ~ 8.0, respectively. Combining the data, we calculate the dielectric constant *ε_r_*(*f*) under different water content *f_w_* according to the Maxwell–Garnett equation. Selected data for several hydrogels are shown in Figure 4B–D corresponding to *f_w_* = 0.383, 0.448, and 0.633, where the symbol Δ represents the difference between the theoretical and measured data in the low and mid-frequency range.

If the hydrogels are regarded as the composites of polymer phase and ice phase, the measured data (black open square curves) should be very similar to the theoretical data (red open circle curves) from Equation (2). However, by comparing the experimental and theoretical data of all samples, it is found that there are big differences for all the hydrogels of different *f_w_*. First, the results for all samples show that the measured dielectric data are much larger than those derived from theoretical calculations. In particular, the dielectric constant at low and medium frequencies may be an order of magnitude larger than the predicted value. This great difference is due to the dielectric relaxation within the interfacial layer, which can be attributed to the high density of polar molecules. Second, it can be seen that the dielectric constant at intermediate frequencies increases with increasing *f_w_*.

These dielectric features exhibited by covalent CS-based hydrogels are similar to those of our previous non-covalent polyacrylonitrile (PAN)-based hydrogels [23]. However, there is still a big difference, which is mainly manifested in the stronger frequency dispersion of the PAN-based hydrogels in the low and mid-low frequency range. Meanwhile, the dielectric relaxation strength (dielectric value) of the interfacial layer of the covalent CS-based ice-hydrogels is about an order of magnitude smaller than that of the non-covalent PAN-based ice-hydrogel, at least in the mid-frequency range [23]. The huge differences in the dielectric data of the two types of hydrogels are mainly due to their different compositions. The CS-based hydrogels are covalently cross-linked, and the density of polarizable electric dipoles in the interfacial layer is comparatively low, while PAN-based hydrogels are predominately by non-covalent interactions including dipole-dipole and hydrogen bonding, and the density of polarizable electric dipoles within the interfacial layer is relatively high [23]. Therefore, it is very necessary to study the interface of covalently bonded hydrogels because it complements and completes the knowledge of the hydrogel interface.

In our non-covalent PAN-based hydrogels research, we used the method of measuring ice-hydrogels to well exclude the EP effect to obtain dielectric data from the interface [23]. In this work, we followed this line, demonstrating that these dielectric data come mainly from polar molecules or polar electric dipoles in the interfacial layer of the polymer and ice phases presented in Figure 4.

### 2.3. Overall Dielectric Relaxation

**Figure 5:**
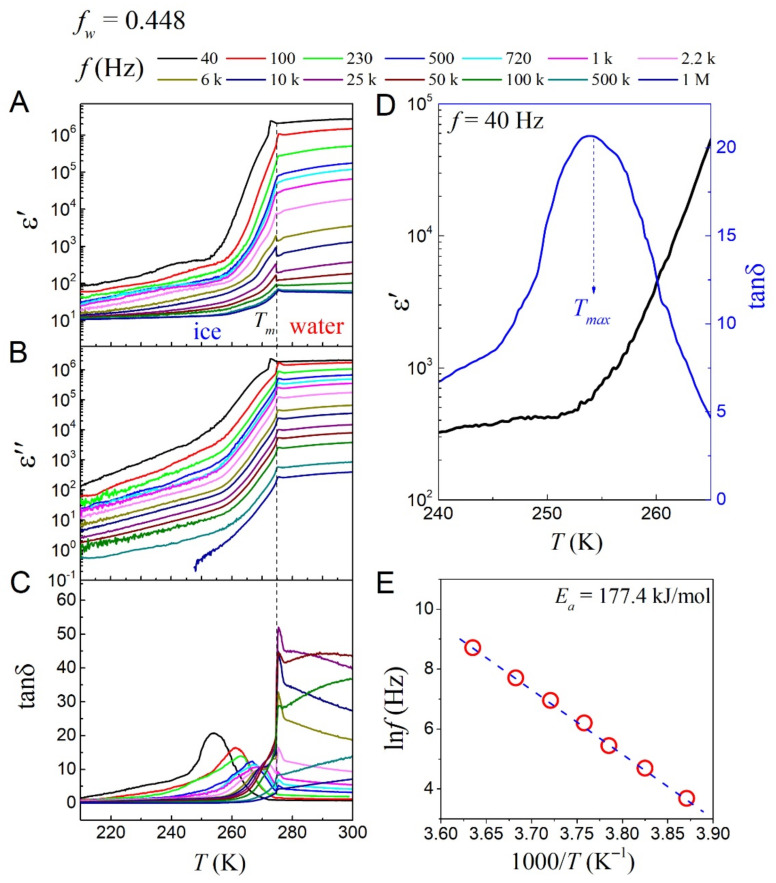
(**A**–**C**) Measured the real part of permittivity *ε*’, imaginary part of permittivity *ε*”, and loss tan*δ* as a function of temperature *T* at several frequencies, respectively, which are the hydrogels (*f_w_* = 0.448) data measured from the heating program starting at 200 K. For more clarity, the measured *ε*’(*T*) and tan*δ* (*T*) data at *f* = 40 Hz are shown in (**D**). (**E**) Calculated the activation energy *E_a_* by the Arrhenius equation and evaluated the relationship between *T_max_* and *f*.

The dielectric relaxation spectra of ice-hydrogels with different samples were measured, and we used these data to analyze the dielectric relaxation behavior of electric dipoles or polarized molecules in the interface layer. The DRS measurement results of the CS-based ice-hydrogel with *f_w_* = 0.448 are shown in Figure 5A–C. The measured dielectric real part *ε*’(*T*) and imaginary part *ε*”(*T*) of the hydrogel during heating as a function of *T* are presented in Figure 5A,B, respectively, where several values of frequency *f* are chosen. There are several main features that should be emphasized. Firstly, the dielectric data is divided into two parts including the water-hydrogels and ice-hydrogels, based on the melting point (*T_m_* ~ 0 °C) of ice. Secondly, when *T* > *T_m_*, the ice-hydrogels melt into the water-hydrogels. Since the influence of the EP effect from the dielectric signals of the hydrogels themselves are completely submerged, this is not the issue for our study. Thirdly, we are concerned with the dielectric real part (*ε*’(*T*)) at *T* < *T_m_*. On the one hand, the *ε*’(*T*) shows an increasing trend with temperature, which is the thermal effect of the dipole inside the dielectric layer. On the other hand, the *ε*’(*T*) reveals strong scattering with frequency at low frequencies and weak realizations at high frequencies.

The dielectric loss (tan*δ*) is also an important parameter in response to dielectric relaxation. The tan*δ* (*T*) as a function of temperature (*T*) is presented in Figure 5C, which clearly shows the single-peak dependence of temperature on the peak position of the tanδ (*T*). There is a clear shift in the loss peak with increasing frequency in the mid- and low-frequency regions, which is evidenced by the strong dielectric frequency dispersion. In the high frequency range, as the temperature is close to *T_m_*, the dielectric response signals are submerged by the EP effect. To better investigate the relaxation characteristics, the dielectric real part (*ε*’(*T*) and loss (tan*δ* (*T*)) data are presented in Figure 5D. The *ε*’(*T*) increases sharply with increasing temperature, and tan*δ* (*T*) shows a wide envelope peak. The linear *ln*(*f*) ~ 1/*T_max_* relation is shown in Figure 5E. This relationship can be described by the Arrhenius equation [29]:

(2) $$f=f_{0}\cdot \exp(-E_{a}/k_{B}T_{\max}),$$

where *T*_max_ is the peak position of the tan*δ* (*T*), *f*_0_ represents the Arrhenius constant, *E_a_* is the dielectric activation energy, and *k_B_* is the Boltzmann constant. The best result by fitting is *E_a_* ~ 177.4 kJ/mol, which is the dielectric activation energy associated with the interfacial layer.

We further show the DRS data for hydrogels with *f_w_* = 0.519 and *f_w_* = 0.694 presented in Supplementary Figure S1. The dielectric relaxation behavior exhibited by these ice-hydrogels samples is similar to that of the ice-hydrogels with *f_w_* = 0.448. According to the Arrhenius equation, the activation energies of the ice-hydrogels with *f_w_* = 0.519 and the ice-hydrogels with *f_w_* = 0.694 are *E_a_* ~ 184.6 kJ/mol and *E_a_* ~ 190 kJ/mol, respectively. It is not difficult to find that the activation energy values of ice-hydrogels with different *f_w_* are very close, which may indicate that the activation energy value is independent of *f_w_*.

### 2.4. Activation Energy and Discussion

**Figure 6:**
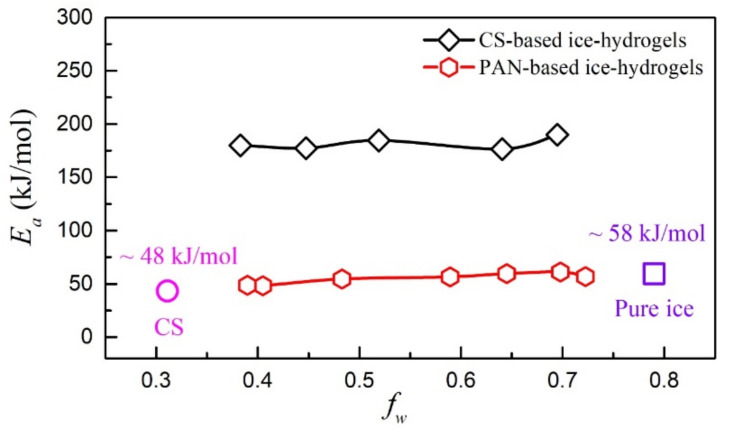
Activation energy (*E_a_*) as a function of water contents (*f_w_*) for covalent chitosan (CS)-based ice-hydrogels and non-covalent polyacrylonitrile (PAN)-based ice-hydrogels.

We measured the activation energy (*E_a_*) of CS-based ice-hydrogels with different *f_w_* plotted in Figure 6, which shows that the *E_a_* ~ 180 kJ/mol, essentially independent of the *f_w_* of the hydrogels. With such a large dielectric activation energy, considering that the hydrogels are a covalent bond structure, this result is physically reasonable. The activation energy *E_a,p_* ~ 48 kJ/mol of CS polymer at low temperature [30] and the activation energy value of pure ice phase *E_a,w_* ~ 58 kJ/mol [31] are marked in Figure 6, respectively. The activation energy value of the CS-based ice- hydrogels is much larger than that of the CS polymer and pure ice phase, which indicates a strong coupling effect of the dipole at the interfacial layer. The large activation energy values of covalently bonded hydrogels are as predicted in our previous work.

We also plot the activation energies of non-covalent polyacrylonitrile (PAN)-based ice-hydrogels with different *f_w_* in Figure 6, which is compared with the data for covalent hydrogels. The activation energy of non-covalent ice-hydrogels has been reported to be roughly 50–60 kJ/mol. It is not difficult to find that there is a large difference between the activation energy values of the covalent CS-based ice-hydrogels and the non-covalent PAN-based ice-hydrogels. This is mainly related to the chemical composition of the hydrogels. CS-based hydrogels are covalently cross-linked and have high dielectric activation energy, which suggests a strong coupling effect of the dipole at the interfacial layer. In contrast, PAN-based hydrogels are non-covalently cross-linked and have low dielectric activation energy, manifesting as weak coupling effect of dipoles at the interfacial layer.

## 3. Materials and Methods

### 3.1. Sample Preparation

The synthesis of CS-based hydrogels has been described in previous reports, and we only briefly describe it here. The synthetic reaction diagram of hydrogels is shown in Figure 2, which is consistent with the information provided in [25].

For the synthesis of CS-based hydrogels, the 1% acetic acid solution was first prepared so that the CS can quickly be dissolved in the solution. Then, 1.0 g CS powder (Aladdin, Shanghai, China) was added to the solution of acetic acid (Aladdin, Shanghai, China) and continuously stirred until it was completely dissolved, forming the CS solution that was stirred at 60 °C. Subsequently, 2 mL 0.1 g/mL Ammonium persulfate (APS) (Aladdin, Shanghai, China) aqueous solution was put into the CS solution. After stirring for 30 min, 14.4 g acrylic acid (AA) (Aladdin, Shanghai, China) and 0.2 g N,N’-Methylenebis (acrylamide) (MBA) (Aladdin, Shanghai, China) were added to the CS solution.

After sufficiently stirring the solution, which was static at 60 °C for four hours so that the synthesis process could be fully carried out, the hydrogel precursor was successfully composed and cooled naturally to room temperature. The hydrogels were immersed in a large amount of pure water. Subsequently, the immersed water was replaced every 24 h and this process was repeated six to seven times to eliminate those non-reacted monomers in the hydrogels. Ultimately, the hydrogels were placed in a container filled with water for preservation.

### 3.2. Structural Characterizations

It is well known that the water content of the hydrogels affects their properties and functions. In general, the equilibrium water content is used to represent the water content of the hydrogels. Typically, estimation of equilibrium water content in hydrogels is measured by the insoluble fraction in dried samples. However, our previous interfacial work on hydrogels has demonstrated that employing volume percentage to represent the equilibrium water content seemed to be more reasonable [23]. Therefore, in this work, we will continue to apply the equilibrium water content in volume percent with the corresponding parameter *f_w_*:

(3) $$fw=\frac{m_{w}/\rho_{w}}{m_{w}/\rho_{w}+m_{p}/\rho_{p}},$$

where, *m_w_* is the mass of water in the hydrogel, *m_p_* is the mass of dry hydrogels, *ρ_w_* represents the density of ice, and *ρ_p_* denotes the dry hydrogel’ density, i.e., *ρ_p_* = 1.085 g/cm.

Direct imaging of the morphological structure of CS-based wet-hydrogels was completed using an optical camera. The microstructure of the wet-hydrogel samples was imaged using the Zeiss optical microscope (SEM, Imager. M1m, Carl Zeiss AG, Jena, Germany), while the microstructure of freeze-dried hydrogels was photographed by environmental scanning electron microscopy (ESEM) with Quanta 200 instrument manufactured by FEI (Hillsboro, OR, USA) in the United States. To obtain the microstructure of the wet-hydrogels, we needed to immerse the wet-hydrogels in liquid nitrogen for one hour, then rapidly transfer them to the freeze dryer to stay them for 3–4 days, and eventually transfer them to the ESEM sample stage for observation. For more detailed operation procedures, please refer to Ref. [23].

To measure the chemical bonding of CS powder, AA liquid and dried CS-based hydrogels were used. We employed the NEXUS 870 infrared spectrometer instrument made by NICOLET (Madison, WI, USA) in the United States. Here, the FTIR data for CS and AA respectively are taken as reference. Before the FTIR measurement, the wet-hydrogels were dried in an oven at 70 °C, and then the samples were placed in a mortar to grind into powder to press into a transparent sheet with a table press for Fourier transform infrared (FTIR) measurement. This operation is suitable for CS powder, but not for AA because it is liquid. Typically, liquid samples are prepared by placing one or two drops on a glass plate to form liquid films. The covered wavelength range for the FTIR testing is from 4000 to 400 cm^−1^ with a detecting resolution of 2.0 cm^−1^.

The crystalline state of the as-prepared hydrogels was measured employing a D8 Advance XRD instrument of Cu Kα radiation (λ = 0.15418 Å) made by Bruker in Germany. The instrument parameters were set to voltage 40 kV, current 40 mA, step size 0.02°, and testing time per step 0.8 s. CS powder and dried CS/PAA hydrogels were tested in coupled Two Theta/Theta mode of XRD ranging from 5 to 60°. The sample stage remained stationary during the test.

In addition, a DSC-200F3 instrument was used to analyze CS-based hydrogels, which was made by Netzsch-Gerätebau GmbH in Selb, Germany. Five identical hydrogel samples were characterized by selecting heating and cooling rates of 10 K/min, 15 K/min, 20 K/min, 25 K/min, and 30 K/min, respectively, and the temperature *T* range of −80 °C to 25 °C.

### 3.3. Electrical Measurements

In this work, the most important experiment was the DRS measurements. For the dielectric measurements, the wet-hydrogels and pure ice were cut into thin plates of 4.0 mm × 4.0 mm × 1.0 mm in dimensions. The gold foils serve as electrodes for parallel plate capacitors, whose size is 4.0 mm × 4.0 mm × 0.1 mm. The CS-based hydrogels were frozen to the ice-hydrogel state for DRS measurements.

The hydrogels contain a large amount of water, and the ice phase of ice-hydrogel will gradually evaporate during long DRS measurements at low temperatures, thus affecting the measurement results. To avoid evaporation, the hydrogels capacitors were sealed in plastic during the operation of the cooling/heating procedure, also making sure that the contact between the samples and the temperature console was good and that the cooling/heating rate was kept slow enough (0.5 K/min).

The DRS measurements of ice-hydrogels were performed utilizing the HP 4294A impedance analyzer (Agilent, Santa Clara, CA, USA) with the frequency range of 40 Hz–1.0 MHz, and the AC voltage of 0.5 V. Notably, all DRS measurements were performed in order of heating from 200 to 300 K. In this case, the sealed hydrogels were slowly cooled down to 200 K at a rate of 0.5 K/min without any electrical bias.

## 4. Conclusions

CS-based ice-hydrogels with covalent bonding structures were investigated by microstructural and chemical bonding characterization. We focused on the dielectric relaxation spectra (DRS) of ice-hydrogels with different water contents (*f_w_*), avoiding the severe EP effect in the water-hydrogels state. The results of DRS measurements of ice-hydrogels permit us to fully investigate the polymer-water interfacial layer and the role it may play in the properties already exhibited by the hydrogels. The polymer-water interfacial layer in this series of CS-based ice-hydrogels with dielectric relaxation behavior exhibits a significant contribution, suggesting that the dielectric response was mainly from the interfacial layer rather than the polymer phase and the ice phase. Meanwhile, the measured DRS data revealed similar activation energies (*E_a_* ~ 180 kJ/mol) for the dielectric relaxation in ice-hydrogels with different *f_w_*. This value is much larger than that of the polymer phase and the ice phase alone, indicating a strong coupling of electric dipoles at the interfacial layer of covalently bonded hydrogels. We have studied the interface of covalent ice-hydrogels in this work, which enables the reader to reconnect with the importance of the hydrogels interface. Nowadays, hydrogels are in full swing as stretchable electronic devices and soft machines, etc., in which the interface of hydrogels plays an important role in these functional devices. Our research on the interface of covalent ice-hydrogels will provide help for researchers in device design and device operation failures.

## Data Availability

Not applicable.

## Supplementary Materials

The following supporting information can be downloaded at: https://www.mdpi.com/article/10.3390/gels8070409/s1.
