# Supplementary material for: Significant Interfacial Dielectric Relaxation of Covalently Bonded Ice-Hydrogels
Source: Gels. 2022 Jun 28;8(7):409. doi: 10.3390/gels8070409 (PMC9322482; doi:10.3390/gels8070409)
Supplement: Supplementary file 1 [file gels-08-00409-s001.zip › gels-1772051-supplementary.pdf]

gels

MDPI

Article

Significant Interfacial Dielectric Relaxation of Covalently Bonded Ice-Hydrogels

Yongqiang Li [1,*], Liufang Chen [1], Chuanfu Li [1], Lin Lin [2], Zhibo Yan [1,*] and Junming Liu [1]

[1] Laboratory of Solid State Microstructures, Nanjing University, Nanjing 210093, China; lfchen502@163.com (L.C.); lichuanfu_com@126.com (C.L.); liujm@nju.edu.cn (J.L.)

[2] Department of Applied Physics, College of Science, Nanjing Forestry University, Nanjing 210037, China; llin@njfu.edu.cn

* Correspondence: yongqgl@126.com (Y.L.); zbyan@nju.edu.cn (Z.Y.)

Supplementary Materials

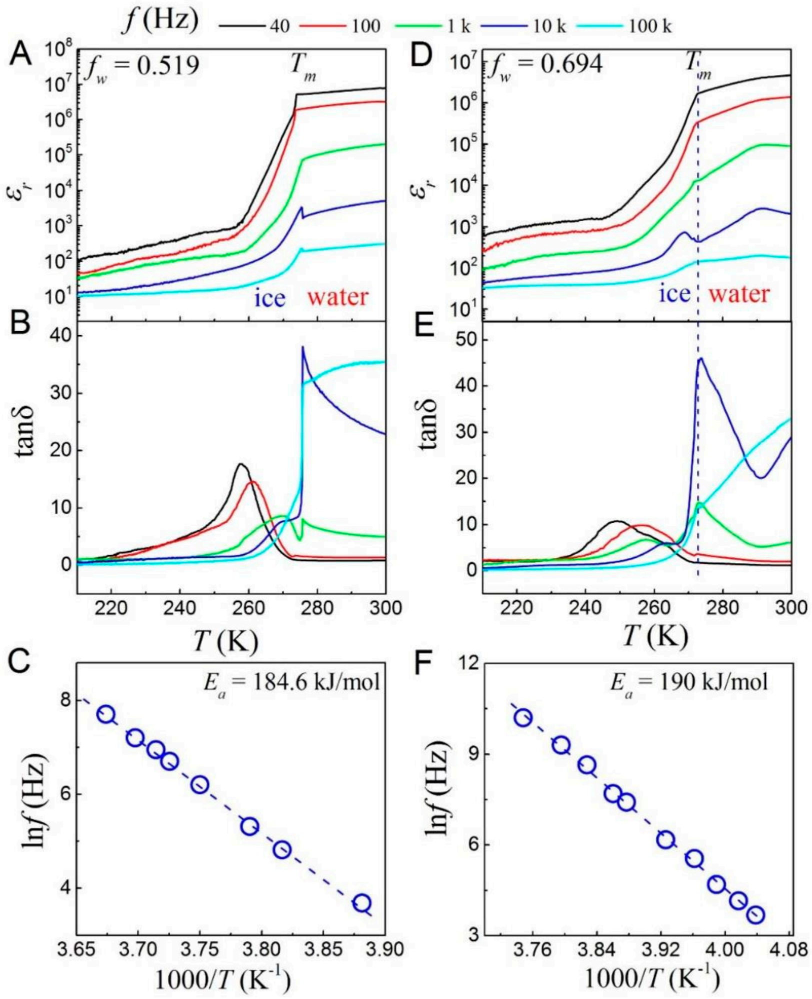

Figure S1. (**A**) and (**D**) measured dielectric constant ε_r as a function of temperature T; (**B**) and (**E**) measured loss tanδ as a function of temperature T function, they are all measured data from the heating program at several frequencies starting at 200 K. (**C**) and (**F**) evaluated T_{max} versus f, from which the activation energy E_a was calculated by the Arrhenius equation. Figures (**A**), (**B**) and (**C**) are hydrogels with f_w = 0.519; Figures (**D**), (**E**) and (**F**) are hydrogels with f_w = 0.694.
